# Sleep Treatments in Disorders of Consciousness: A Systematic Review

**DOI:** 10.3390/diagnostics12010088

**Published:** 2021-12-31

**Authors:** Martina Cacciatore, Francesca G. Magnani, Matilde Leonardi, Davide Rossi Sebastiano, Davide Sattin

**Affiliations:** 1 UOC Neurologia, Salute Pubblica, Disabilità, Fondazione IRCCS Istituto Neurologico Carlo Besta, 20133 Milan, Italy; martina.cacciatore@istituto-besta.it (M.C.); matilde.leonardi@istituto-besta.it (M.L.); 2 Unità di Neurofisiopatologia, Fondazione IRCCS Istituto Neurologico Carlo Besta, 20133 Milan, Italy; davide.rossi@istituto-besta.it; 3 IRCCS Istituti Clinici Scientifici Maugeri di Milano, 20138 Milan, Italy; davide.sattin@icsmaugeri.it

**Keywords:** sleep, sleep disorders, sleep–wake cycle, minimally conscious state, vegetative state, DOC

## Abstract

Sleep disorders are among the main comorbidities in patients with a Disorder of Consciousness (DOC). Given the key role of sleep in neural and cognitive functioning, detecting and treating sleep disorders in DOCs might be an effective therapeutic strategy to boost consciousness recovery and levels of awareness. To date, no systematic reviews have been conducted that explore the effect of sleep treatments in DOCs; thus, we systematically reviewed the existing studies on both pharmacological and non-pharmacological treatments for sleep disorders in DOCs. Among 2267 assessed articles, only 7 were included in the systematic review. The studies focused on two sleep disorder categories (sleep-related breathing disorders and circadian rhythm dysregulation) treated with both pharmacological (Modafinil and Intrathecal Baclofen) and non-pharmacological (positive airway pressure, bright light stimulation, and central thalamic deep brain stimulation) interventions. Although the limited number of studies and their heterogeneity do not allow generalized conclusions, all the studies highlighted the effectiveness of treatments on both sleep disorders and levels of awareness. For this reason, clinical and diagnostic evaluations able to detect sleep disorders in DOC patients should be adopted in the clinical routine for the purpose of intervening promptly with the most appropriate treatment.

## 1. Introduction

After severe Acquired Brain Injury (sABI), certain patients may end up with prolonged/chronic Disorders of Consciousness (DOCs), characterized by alterations of self and/or environmental awareness. Chronic DOCs include the Vegetative State (VS; also known as Unresponsive Wakefulness Syndrome (UWS)), a condition of vigilance unconsciousness, and the Minimally Conscious State (MCS), an altered state of consciousness in which behavioral signs of awareness, albeit reduced and fluctuating, are preserved [1]. Several methods are currently used to evaluate the responsiveness of DOC patients, including behavioral scales (e.g., the revised coma recovery scale [2]) and instrumental tools such as Positron Emission Tomography (PET), functional Magnetic Resonance Imaging (fMRI), and an electroencephalogram (EEG) [3,4,5]. The use of such instrumental tools can be more informative than relying only on behavioral signs of responsiveness, as certain patients may show brain functional activity while being behaviorally unresponsive [6]. Despite the use of multimodal assessment, which has allowed for better framing of the functional status of patients with DOC, one of the major remaining challenges in clinical practice is finding ways to support the recovery of consciousness, increased awareness, and (residual) cognitive functioning in patients.

Since the disconnection of the cortico-cortical and cortico-thalamo-cortical pathways has been recognized as one of the underlying causes of DOC [7], some treatments used to boost consciousness recovery are represented by interventions acting on different neurotransmitter systems to restore the networks’ functionality. Specifically, it has been hypothesized that DOCs are characterized by dysregulations involving excitatory wake-promoting and inhibitory sleep-promoting neurotransmitters which are involved in maintaining efficient connections between high-order cortical areas and sub-cortical structures [7]. This view is in line with what has been theorized on the nature of consciousness. For instance, the Global Neuronal Workspace (GNW) [8,9] hypothesizes that consciousness is determined by information broadcasting through the global workspace represented by high- and low-order areas including prefrontal, associative cortices, thalamus, and subcortical regions. Specifically, these neural structures would be interconnected in a feed-forward fashion so as to make the information available to the system. Similarly, Integrated Information Theory (IIT) is grounded on two key properties of consciousness: integration, and differentiation (or segregation) [10]. Specifically, as pointed out by Tononi, “*consciousness corresponds to the capacity [of a system] to integrate information*” [10] (p. 3). Information integration depends on thalamo–cortical dynamics in terms of both short- and long-range excitatory/inhibitory connections [11]. Despite this remaining only a hypothesis, DOCs could be due to insufficient network integration and segregation. A reduction of both integration and segregation has been empirically described when an alteration of the diurnal sleep–wake cycle intervenes. For instance, both resting-state fMRI and EEG studies comparing network dynamics pre- and post- sleep deprivation on healthy individuals showed changes at the level of the brain networks’ functional connectivity, indicating a reduction of information integration [12,13,14] as well as a reduced segregation of networks highly segregated under regular sleep–wake cycle conditions [12], suggesting that “*the preservation […] of an optimal network architecture for information processing is affected by sleep […]*” [14] (p. 41).

Sleep usually plays a pivotal role in restoring and preserving the brain and the whole body’s health [15,16]. Indeed, sleep is strongly implicated in brain development and cortical maturation favoring the production of the myelin sheath [17] and new synaptic connections [18], as well as enhancing learning and memory consolidation [19,20], and even reducing the DNA damage in neurons [21]. Furthermore, it is well known that the alteration of the normal sleep–wake cycle correlates with a decrease in the cognitive performance of healthy subjects [22]. Neuroimaging studies on sleep-deprived subjects showed lower functional brain activation during active paradigms relying on attention [23] and working memory [24], as well as a decrease in brain functional connectivity within the key regions of the default mode network and the dorsal attention network during rest [25,26].

The above-mentioned evidence, along with the high probability of patients with DOC to develop sleep disorders, suggest that analyzing patients’ sleep performance could have clinical relevance from diagnostic and therapeutic points of view. Abnormalities of circadian rhythms, the sleep–wake cycle, the duration of sleep, and sleep patterns have been observed in DOC patients [27,28,29]. Some studies have suggested a strong relationship between sleep and consciousness even during the acute/subacute phase in sABI patients, as proved by a concomitant recovery of consciousness and amelioration of residual cognitive performances with the return to an almost physiological sleep–wake cycle in DOC patients [30,31,32]. Furthermore, the restoration of a patterned sleep in DOC patients has highlighted the role of sleep as a positive prognostic marker for a good clinical outcome following a traumatic brain injury [32,33,34]. The presence of physiological sleep is likely related to better recovery because it depends on the integrity of the brain circuits that support consciousness and the functional engagement of networks during wakefulness [35,36]. However, it is not simple to detect and classify sleep disorders in DOC patients as suggested by international classifications [37], even if insomnia, abnormal sleep–wake cycles, sleep apnoea, and parasomnias have been described following sABI [38,39,40].

So far, in this challenging context, the treatment of sleep disorders in patients with DOC remains “pioneering”, although early evidence suggests that therapeutic strategies, able to ameliorate sleep and sleep–wake cycles, may improve recovery in DOC patients [36]. In this line, the recent European Academy of Neurology guidelines on comas and DOCs [41] does not detail which sleep treatments must be applied in DOCs, while emphasizing the role of sleep assessments in patients with DOC for diagnostic purposes. Indeed, it is currently unclear if sleep disorder treatments must be applied, managed, and “customized” for the specific needs of DOC patients and, although some systematic reviews have focused on pharmacological and non-pharmacological treatments of sleep disorders in patients with acquired brain injury [42,43], to date, none have focused on DOC patients.

With the aim of filling this gap, we conducted a systematic literature review to investigate current published studies that intervened, pharmacologically or non-pharmacologically, to improve sleep and/or treat sleep disorders in patients with DOC.

## 2. Materials and Methods

The present systematic review followed the Preferred Reporting Items for Systematic Reviews and Meta-Analyses guidelines (PRISMA, [44]) to search and extract eligible studies.

### 2.1. Search Strategy

Relevant studies were identified by enquiring the following electronic databases: MED-LINE/Pubmed, EMBASE, Scopus, Web of Science, and Cinahl Complete. We adapted the search strategy for each database using the Patient, Intervention, Comparison, Outcome (PICO) framework without specifying the types of comparisons (see Supplementary Materials for terms combinations).

The publication dates were set from the beginning of 2011 until February 2021, and all the searches were limited to studies written in English and Italian languages. Only studies on humans were considered. Given the explorative aim of the present review, no limit on article type was predetermined.

The reference list of the retrieved studies was also assessed to identify further studies respecting the selection criteria.

The results obtained from each database were exported to web-based bibliographic management software (Mendeley; https://www.mendeley.com, accessed on 1 March 2021), and duplicate deletion was performed. Moreover, all the records were imported in a tailored Excel spreadsheet including the title, abstract, and record information for each article.

### 2.2. Selection Criteria

Studies were eligible if they met all the following criteria: (1) studies on patients with a diagnosis of DOC based on clinical evidence; (2) the presence of sleep disorders; (3) description of the treatment for sleep disorders or interventions to restore sleep quality.

All the main categories of sleep disorders identified by ICSD-3 [37] were included. Likewise, both pharmacological and non-pharmacological (including the use of specific devices) treatments were included.

### 2.3. Screening

After duplicates deletion, the search results of each database were independently screened by two expert raters.

In the first step, two raters (Rater #1: F.G.M.; Rater #2: M.C.) screened the articles by title and abstract, using the following assessment scale: 0 = the article did not meet the inclusion criteria (e.g., it was specified that patients were not diagnosed with DOCs in the title and abstract); 1 = the article matched the inclusion criteria; 2 = the reviewer doubted the inclusion of the article, which required a full-text assessment (e.g., if there were any moderate or severe Traumatic Brain Injury diagnoses, but without specification for a DOC).

The agreement between the two raters was computed (i.e., Cohen K analysis [45]). In cases of discrepancy between rater #1 and rater #2 on inclusion of a specific article, a third rater (rater #3: D.S.) with senior experience in systematic reviews and DOC patients screened the article to solve this discrepancy.

In the second step, the same raters as in the first step (rater #1 and rater #2) analyzed the full text of all the studies included after the first step. Then, a rating scale with a binary code (i.e., 0 = excluded, 1 = included) was used. Both the raters agreed upon which studies met the inclusion criteria. Furthermore, in this step, the complete reference list of each article (including the review articles) was read to identify further eligible studies to be added.

### 2.4. Data Extraction

Two raters independently extracted data from the included studies, using a custom-built Excel datasheet. From each study, the following data were extracted: study type, sample size, demographic characteristics, diagnosis and etiology of the sample, presence of control group or control condition, type of sleep disorder, treatment and any concomitant therapies, treatment duration, outcome measures, and statistics when available.

There was no disagreement on the data extraction between the two raters (#1, #2) which would have been resolved by the intervention of the third rater (#3).

## 3. Results

### 3.1. Literature Search Results

The PubMed, EMBASE, Scopus, Web of Science, and Cinahl Complete database searches identified 330, 932, 576, 383, and 46 studies, respectively, leading to a total of 2267 articles. The automatic removal of duplicates resulted in 1704 articles.

**Figure 1:**
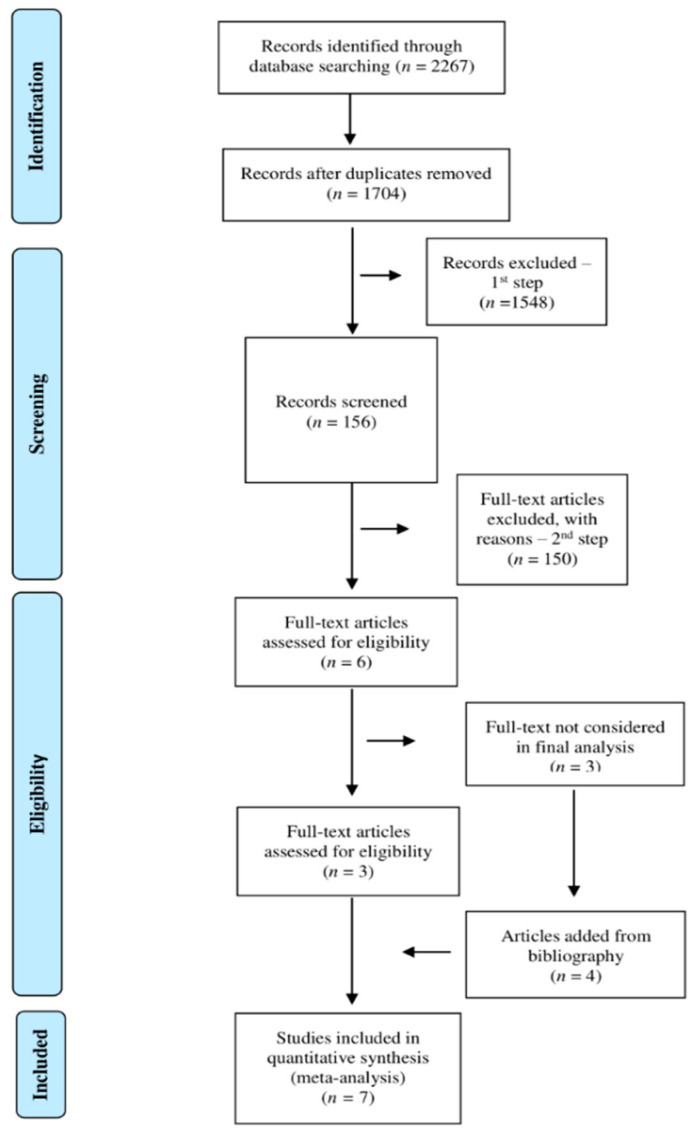
Flow diagram of this systematic review. Adapted from [44].

During the first step for title and abstract selection, raters #1 and #2 agreed upon including 132 studies in the second step. Furthermore, rater #3 screened 262 studies for which there was no agreement between rater #1 and #2, including 24 studies in the second step. Consequently, 156 full texts were assessed in the second step (see Figure 1).

The Cohen’s Kappa value for inter-raters agreement in the first step was 0.79, meaning that there was a ‘Substantial’ concordance between the raters.

After full texts analysis in the second step, only six articles met the inclusion criteria for this systematic review. There were two main reasons for exclusion: (i) studies on patients without DOC; (ii) lack of specific treatment for sleep disorders or interventions to restore sleep. Three of the six articles were not included in the final analyses as they were review articles, while four articles were added from their reference lists. Therefore, seven studies were eligible for analysis in the systematic review (Figure 1).

### 3.2. Studies’ Design of the Articles Analyzed

The majority of the included studies were case reports (*n* = 5); [46,47,48,49,50], one study adopted a cross-sectional design [51], and one was a retrospective pilot study [52].

### 3.3. Clinical Populations Considered in the Included Studies

Overall, the included studies involved 36 DOC patients (25 males) suffering from traumatic (*n* = 13) and non-traumatic (*n* = 23) ethiological events. Specifically, 15 patients were diagnosed with MCS, 1 emerged from MCS (eMCS), and 20 were diagnosed with VS. The time from injury ranged from 2 to 252 months (mean ± SD: 29.12 ± 43.6 months).

### 3.4. Sleep Disorders and Treatments

Two articles focused on treatments for sleep-related breathing disorders [49,50], namely central apnea and obstructive apnea, respectively. The other five included articles examined different treatments to improve circadian rhythm regulation and the sleep–wake cycle, including extreme fragmentation of nocturnal sleep and excessive sleepiness (*n* = 3; [47,51,52]) and alterations in sleep dynamics (*n* = 2; [46,48]).

As for treatments, three studies adopted pharmacological interventions [47,49,52], one study described an environmental intervention [51], one study relied on the use of a device to treat sleep disorder [50], while two studies focused on the use of brain stimulation [46,48]. In what follows, interventions are presented for each category of sleep disorders (i.e., sleep-related breathing disorders and sleep rhythm dysregulation); see also Table 1 for the main features of the studies included in this review.

#### 3.4.1. Treatments for Sleep-Related Breathing Disorders

Silva et al. [50] described a severely brain injured MCS patient who presented obstructive sleep apnea treated with Positive Airway Pressure (PAP). After 35 weeks of treatment, the authors detected an improvement in sleep efficiency and architecture, along with ameliorations in motor and cognitive functioning (see Table 1 for more details).

Differently, Locatelli et al. [49] described a pediatric MCS patient who developed central sleep apnoea possibly due to an increased Intrathecal Baclofen (ITB) dosage (from 450 μg/die to 600 μg/d) administered for spasticity. In this case, the central sleep apnoea was treated through Baclofen tapering (reduced to 100 μg/day) leading to a significant improvement of respiratory patterns as detected by polysomnography. Furthermore, improvements in motor and cognitive functioning were reported as anecdotal evidence, lacking any kind of quantitative behavioral data [49].

#### 3.4.2. Treatments for Sleep Rhythms Dysregulation

Two studies adopted a pharmacological treatment with Modafinil [47,52] for sleep rhythm normalization. Specifically, in a retrospective pilot study [52] on 24 patients diagnosed with VS or MCS, Modafinil in addition to standard therapies had beneficial effects in treating excessive sleepiness and improving cognition. A recovery of the patients’ consciousness level, assessed through the CRS-r and the total Wessex Head Injury Matrix (WHIM), was observed after Modafinil administration. Specifically, a significant difference was found in the total WHIM score between pre-intervention (meantotal score = 7.39) and post-intervention (meantotal score = 10.94; *p* = 0.002); seven patients changed from VS to MCS, and four patients regained full consciousness.

Similarly, in a study by Formica et al. [47], the use of Modafinil in addition to Baclofen, Delorazepam, and Melatonin led to a partial restoration of circadian rhythms with a considerable increase of sleep–wake periods in a VS patient. Furthermore, there was an improvement in the patient’s awareness, as detected by several outcome measures, including the DRS (a total score decrease from 19 to 14) and the Level of Cognitive Functioning Assessment Scale (LOCFAS; a level increase from 2 to 3).

Only one study described the effects of an environmental intervention on circadian rhythm regulation on 8 patients [51]. Specifically, the authors demonstrated the effectiveness of Bright Light Stimulation (BLS) in treating circadian rhythmic sleep disorders. Although the behavioral data collected through CRS-r correlated with a physiological index (i.e., body temperature) used as a proxy for circadian rhythm regulation, the study did not report any evidence of efficacy directly related to the treatment. However, the authors stated that three out of the eight patients enrolled in the study showed behavioral and awareness improvements after the intervention, changing their diagnoses from VS to MCS/eMCS.

Finally, two studies using Central Thalamic-Deep Brain Stimulation (CT-DBS) [46,48] described the progression of the same MCS patient over 6 years. CT-DBS treatment resulted in improvements in their sleep pattern, with a normalization of sleep dynamics; the authors reported an increase in the frequency of sleep spindles (spindles with high frequency are related with a good outcome in the subacute phase following sABI [53]) during Non-Rapid Eye Movement-2 (NREM-2), and during Slow Wave Sleep (SWS) stages. However, a disruption in sleep architecture was found one year after the discontinuation of CT-DBS [48]. Moreover, the behavioral examination showed that significantly lower CRS-r scores (mean_total score_ = 9) were observed after cessation of the treatment, although there were no statistical differences in the CRS-r scores between different time points of the follow-up of the patient when treated (mean_total score_ = 11.8).

## 4. Discussion

Although it is recognized that sleep disorders are among the main comorbidities in sABI patients, and especially for those with DOC, and considering the pivotal role that sleep plays on cognitive and neural functions [15,16], no strong evidence emerges on the effect/efficacy of treatments for sleep disorders in relation to the recovery of consciousness and/or of the improvement of cognitive and behavioral functions in this population. This systematic review, therefore, aimed to fill this gap by exploring the literature on pharmacological and non-pharmacological treatments of sleep disorders in patients with DOC.

We found only seven studies focusing on the treatment of sleep disorders in patients with DOC, whose experimental designs were heterogeneous. Indeed, most were case reports [46,47,48,49,50], one study had a cross-sectional design [51], and one was a retrospective study [52]. Despite this null result, we think that the limited number of studies is itself informative, proving treatments of sleep disorders on DOCs are barely explored, at least in the last 10 years. A side note is due here, as during the last 10 years there has been an increase in literature produced on experimental paradigms with DOCs (see, for instance, data in the database by Yaron et al. [54]; https://contrastdb.tau.ac.il/#fMRIResults_Title, accessed on 22 December 2021) indicating how there has been a growing interest in exploring the nature of DOCs while it is likely fewer efforts have been put on exploring the efficacy of treatments for consciousness recovery, especially when they act indirectly on consciousness recovery, as happens in the case of sleep disorder treatments.

The treated sleep disorders in the analyzed articles were heterogeneous as well and limited to two main categories: Sleep-related breathing disorders [49,50], and circadian rhythm sleep–wake disorders [46,47,48,51,52]. The adopted treatments included both pharmacological and non-pharmacological interventions.

Considering pharmacological interventions, Modafinil was found to be adopted to improve the circadian rhythm regulation and to reduce excessive (daily) sleepiness [47,52], while Baclofen tapering was adopted for breathing disorder [49]. Specifically, Modafinil is a dopamine reuptake inhibitor that overrides the expression of orexin, a neuropeptide involved in wakefulness and attention, whose exhaustion appears to be associated with fatigue, narcolepsy, and excessive sleepiness [55]. It is a drug with effectiveness in treating daytime drowsiness associated with OSA [56], narcolepsy [57], and shift work sleep disorder [58]. Modafinil is well tolerated, it has a lower incidence of adverse cardiopulmonary effects than other neurostimulants, and it has a relatively low potential for abuse [59,60]. In addition, this drug is associated with improvements in cognitive performance both in clinical populations [61,62] and healthy individuals, as attested by improved performance in attentive, mnemonic, and fluid intelligence tests after drug administration [63,64,65]. Modafinil administration is also associated with increased activation in the Frontal Parietal Control (FPC) and the Dorsal Attention Network (DAN), and the modulation of functional connectivity of the resting state of networks, including the FPC, the DAN, and the Exstrastriate Visual System [66], suggests that Modafinil, at least in a healthy system, can promote network integration. Consequently, it can be hypothesized that Modafinil administration to DOC patients, while treating sleep disorders, could also promote recovery of consciousness in line with some theories’ hypotheses (i.e., GNW and IIT [8,9,10]).

The use of neurostimulants in the management of patients with DOC is still controversial, and the existing studies are focused more on the role of agents that have a “direct” effect on recovery of consciousness, like Amantadine (a synthetic tricyclic amine with antiviral, antiparkinsonian, and antihyperalgesic activities) or Zolpidem (a benzodiazepine receptor agonist that is used for the treatment of insomnia; see for example [67,68,69,70]), leaving aside analysis on their potential effects on sleep disorders [71]. ITB is, instead, mainly used as a central action treatment to improve spasticity, even in patients with DOC, and few uncontrolled studies and case reports have suggested ITB has a potential role in stimulating recovery of consciousness [72,73]. On the contrary, the study included in the present review attested how ITB dosage increase could provoke sleep breathing disorder which was indeed resolved throughout ITB tapering [49]. Thus, despite its potential effect on consciousness recovery, using ITB at high dosage should be carefully evaluated due to its possible interaction with sleep dynamics.

As for non-pharmacological interventions, we identified PAP as a treatment for breathing disorder [50], while BLS [51] and CT-DBS [46,48] were used as treatments for circadian sleep–wake disorders. In patients with OSA, the efficacy of treatment with PAP in reducing sleep-apnoea is associated with cognitive recovery [74]. Therefore, the analysis of PAP, in terms of tolerance and indirect effectiveness on the consciousness recovery in patients with DOC, should be better evaluated in the near future. Light therapy is a form of natural treatment based on the influence that environmental light has on the biological clock in the suprachiasmatic nuclei of the hypothalamus, which regulates circadian processes [75]. BLS has been effectively used to improve the sleep–wake cycle even in neurodegenerative disorders [76] and could have effects not only on the regulation of circadian rhythms but also on the processes that support sustained attention and awareness, as demonstrated by some studies [77,78,79]. Finally, CT-DBS was used to exploit the key role of the thalamus in regulating the excitation of the forebrain and restarting affected networks or modulating aberrant/desynchronized activity between brain areas, preventing the wide deafferentation of the brain [80]. Furthermore, given the connection between the thalamus and cerebral frontostriatal systems, CT-DBS in DOC patients could influence the restoration of sleep dynamics by facilitating the activation of the fronto-cortical circuit, as supposed by Adams and colleagues [46].

All the studies reported, directly or indirectly, the treatments’ efficacy on sleep disorders. Modafinil had beneficial effects in treating excessive sleepiness by promoting the restoration of circadian rhythms, with a significant increase and stabilization of sleep–wake periods [47,52]. Similarly, BLS had been effectively used for circadian restoration in DOC patients [51]. The data also supposed the efficacy of CT-DBS, indicated by the modification found in the cortical activity, and the restoration of good sleep architecture and of almost physiological NREM-2 and SWS stages of sleep, [46] results which, however, regressed when treatment was discontinued [48]. For sleep-related breathing disorders, PAP showed evidence of efficacy in treating OSA in DOC patients [50], while central sleep apnoea was treated effectively through Baclofen tapering [49].

Although the articles included in this review did not provide strong evidence of efficacy (even considering that we did not find Randomized Controlled Trials), it is worth noting that all the included studies showed behavioral and cognitive improvements, and/or an increased awareness in DOC patients. Furthermore, in two studies, a total of 14 patients changed their diagnosis from VS to MCS or fully recovered consciousness [51,52]. Referring to what is hypothesized by the GNW and the IIT [8,9,10] on the nature of consciousness, the efficacy of some treatments, including Modafinil and CT-DBS, on consciousness recovery is, at least theoretically, more probable than other treatments acting in different ways. Specifically, if one takes the assumption that consciousness recovery is strictly related to the restoration of network integration while preserving the segregation, then treatment efficacy should be found for treatments promoting these mechanisms. On the one hand, as mentioned before, Modafinil seems to promote the activity of high-order areas (fundamental for the top-down modulation taking the GNW as reference theory [8,9]) as well as functional connectivity between different networks, (fundamental for integrating information taking the IIT’s point of view [10]). Similarly, CT-DBS starting from the thalamus promotes information exchange between low- and high-order areas, which is in line with what the GNW hypothesized as being at the base of consciousness [8,9]. As a matter of fact, the studies on CT-DBS [46,48] and Modafinil [47,52] showed awareness improvement in all the treated patients, despite their long-term effects not yet being explored in randomized controlled trials. On the other hand, the efficacy of different treatments, including ITB tapering, PAP, and Bright Light, on consciousness recovery cannot be easily hypothesized taking into account the above-mentioned theoretical background [8,9,10]. The evidence on these treatments only supports the beneficial effect treating sleep disorders has on the level of responsiveness. Taken together, the analyzed studies can only support the assumption that treating sleep disorders could boost consciousness recovery due to the strict link between sleep and consciousness both from a neuroanatomical and functional point of view [30,31,32,35,36]. Future studies, aimed at exploring the effect of sleep disorder treatments in patients with DOC, should better frame the hypotheses underlying their mechanisms from a theoretical point of view. This could help to achieve two aims: (i) providing more evidence on tailored treatments for DOCs, and (ii) contributing to the theoretical debate on the nature of consciousness.

Overall, the treatment of sleep disorders in DOC patients could be promising in the context of fostering awareness recovery. However, the results of our review show that the treatment of sleep disorders in patients with DOC is currently limited. Although the literature recommends intervening on secondary comorbidities [1], including sleep, international guidelines do not directly refer to the treatment of sleep disorders [41,81,82]. Indeed, the European Academy of Neurology guidelines [41] consider sleep as a further diagnostic marker to differentiate VS from MCS, while the American Academy of Neurology guidelines [82] mentions sleep architecture only for its prognostic role. In addition, we think that the time has come to focus on sleep patterns in DOC patients, since treatments for sleep disorders/dysregulation could also have beneficial effects in terms of consciousness recovery. In this respect, we suggest that the implementation of polysomnography in the standard clinical routine could be of great help, since it would allow clinicians to promptly identify not only prognostic and diagnostic markers but also potential sleep disorders that, if treated, could boost the patient’s state of awareness recovery. The lack of polysomnography examinations in standard clinical practice for DOC patients is reflected by the limited variety of disorders described and treated in the studies found by our systematic review (sleep-related respiratory disorders and sleep–wake circadian rhythm disorders), which could be due precisely to the absence in the clinical routine of tests able to detect sleep disorders in this population. Indeed, looking at the studies describing sleep disorders in the broader clinical category of patients with sABI, we found descriptions of other sleep disorders beyond the categories included in this review, such as Periodic Limb Movements and bruxism [38,83]. Data in the literature show that, if neglected, these sleep disorders can lead to several sequelae that could aggravate both the general clinical situation and cognitive functioning of patients, including their risk of developing cardiovascular disease [84], executive function deficiency [85], and increased pain perception [86,87].

Although no studies explored the consequences of the prolonged absence of treatments for sleep disorders in DOC patients, future studies should focus on the best ways to diagnose and treat the main sleep disorders of these patients in a timely manner. This is necessary both in light of the relationship that sleep has with consciousness [29,88,89] and considering the limited cognitive reserve characterizing DOC patients.

### 4.1. Limits

Some limitations must be accounted for. First, we considered only literature from the last 10 years (from the beginning of 2011 until February 2021), thus excluding potential studies focused on the treatment of sleep disorders in DOC patients dated before this. Also, the conclusions of this systematic review could not be generalized because of the limited number of studies found and their heterogeneous experimental designs. Furthermore, in the analyzed studies, there were no control groups, and thus no comparison between treated and untreated groups was possible.

Again, no causal relationship between awareness and sleep restoration could be drawn; at most, a parallel improvement in both awareness and the sleep/sleep–wake cycle was observed. Moreover, the treatment of sleep disorders was not the main aim of some studies, but an effect which appeared during the study. For instance, the aim of the Dhamapurkar et al. [52] study was to evaluate the efficacy of Modafinil on consciousness recovery in DOC patients without a focus on sleep, while Blume et al. [51] aimed to investigate changes in the circadian rhythms of temperature via light stimulation. In both cases, the interventions showed beneficial effects on recovery of awareness, which the authors attributed to the efficacy of the interventions themselves in treating excessive sleepiness by promoting the restoration of circadian rhythms. Nevertheless, no causal relationship can be proven.

Only two articles reported pre- and post-sleep treatment comparison analyses. Specifically, in the study by Gottshall et al. [48], the effectiveness of the treatment was confirmed by statistical comparisons of the electrophysiological parameters of sleep and behavioral improvement pre- and post-treatment; Dhamapurkar et al. [52] reported such a significant improvement in the state of awareness of patients (assessed through the WHIM) that they suggested this was related to the beneficial effect of the treatment (i.e., Modafinil) on excessive daytime sleepiness. The other studies, instead, relied on qualitative outcome data.

### 4.2. Future Directions

Although the data collected from these studies are limited, several suggestions can be made for clinical practice. Given the high comorbidity that DOC has with sleep disorders [1], and the relationship between sleep and the main neuro- and psychophysiological-functions [15,16], it would be appropriate to consider the sleep–wake cycle in future assessments, implementing the use of the polysomnography in the standard clinical routine for the evaluation of DOC patients. Identifying and treating sleep disorders in DOC patients in a timely manner would help to avoid the consequences of alterations in the normal sleep–wake cycle of patients, which can undermine the recovery process from sABI. Evidently, further studies need to test the safety of standard sleep disorder treatments already adopted in sleep medicine in the context of patients with DOC. It is therefore important to promote trials aimed at verifying the efficacy tolerance of sleep disorder treatments, and their possible interactions with other therapies commonly used for patients with DOC. Treatments targeting sleep disorders in DOCs could be one of the responses to the ongoing challenge of finding conditions that can facilitate recovery of consciousness.

## 5. Conclusions

The results of this systematic review draw attention to the importance of treating sleep disorders in DOC patients, while also promoting neurological and awareness recovery. We suggest the importance of more detailed clinical evaluations and diagnostic screening (e.g., polysomnography) in daily clinical practice to diagnose sleep disorders that undermine sleep quality and, most likely, consciousness recovery. However, given the limited number of studies found in the literature, further studies are needed to better quantify how to treat sleep disorders in these patients and the effect this has on consciousness recovery.

## Figures and Tables

**Table 1:** Studies included in the systematic review. The table shows the main features of the studies included in the systematic review.

								Follow Up	
Authors	Patients	Etiology	Sleep Disorder	Intervention	Dose	Treatment Duration	Behavioral Measures	Time	Sleep Measures
Dhamapurkar et al., 2017 [52]	VS = 16 MCS = 8	TBI = 12 n-TBI = 12	Excessive daytime sleepiness	Modafinil	From 100 mg up to 300 mg daily or the maximal tolerated dose	24 weeks (in average)	WHIM; CRS-r	From 4 to 72 weeks	Sleep–wake cycle behavioral charts
Formica et al., 2017 [47]	VS = 1	n-TBI	Circadian sleep disorders	Modafinil (Baclofen, Delorazepam, Melatonin as concomitant therapies)	100 mg bid	4 weeks	DRS; CNCS; LOCFAS	1 day	24-h PSG; presence and features of sleep stages
Blume et al., 2019 [51]	MCS = 4 VS = 3 eMCS = 1	TBI = 1 n-TBI = 7	Cyrcadian rhythms regulation	Habitual light; bright light	HL: below 500 lux at eye level from 7 a.m. to 9 p.m. daily; BL: around 2000 lux at eye level for 1 h three times a day	2 weeks	CRS-r	1 week	n/a
Locatelli et al., 2019 [49]	MCS = 1	n-TBI	Central Sleep Apnea	Baclofen	Initial: 450 μg/d; 1st increase: 600 μg/d; 2nd increase: 700 μg/d; 3th decrease: 650 μg/d; 4th decrease: 100 μg/d	30 weeks and 2 day	n/a	10 weeks	Apnoea hypopnea index; oxygen saturation
Silva et al., 2019 [50]	MCS = 1	n-TBI	Obstructive Sleep Apnea	PAP	Nightly	35 weeks	CRS-r; DRS; FIM	between 10 and 45 weeks	PAP compliance monitoring using propriety software that measures devise usage.
Adams et al., 2016 [46]	MCS = 1	n-TBI	Unusual mixing of sleep features (as revealed by EEG)	CT-DBS		240 weeks	CRS-r	240 weeks	Background EEG activity in awake; presence and features of sleep stages
Gottshall et al., 2019 [48]						48 weeks (discontinuation of therapy)	CRS-r	288 weeks	Background EEG activity in awake; presence and features of sleep stages

Abbreviations: VS = Vegetative State; MCS = Minimally Conscious State; TBI = Traumatic Brain Injury; n-TBI = non-Traumatic Brain Injury; PAP = Positive Airway Pressure; CT-DBS = Central Thalamic-Deep Brain Stimulation; WHIM = Wessex Head Injury Matrix; CRS-r = Coma Recovery Scale- Revised; LOCFAS = Level of Cognitive Functioning Assessment Scale; CNCS = the Coma/Near Coma Scale; DRS = Disability Rating Scale; FIM = Functional Independence Measure; EEG = electroencephalogram; n/a = not available.

## Data Availability

Data available in an accessible repository on request.

## Supplementary Materials

The following are available online at www.mdpi.com/article/10.3390/diagnostics12010088/s1, Supplementary Materials: search strategy.

## References

[1] Giacino J.T., Fins J.J., Laureys S., Schiff N.D. (2014). Disorders of consciousness after acquired brain injury: The state of the science. Nat. Rev. Neurol..

[2] Giacino J.T., Kalmar K., Whyte J. (2004). The JFK Coma Recovery Scale-Revised: Measurement characteristics and diagnostic utility. Arch. Phys. Med. Rehabil..

[3] Gosseries O., Pistoia F., Charland-Verville V., Carolei A., Sacco S., Laureys S. (2016). The Role of Neuroimaging Techniques in Establishing Diagnosis, Prognosis and Therapy in Disorders of Consciousness. Open Neuroimag. J..

[4] Gosseries O., Di H., Laureys S., Boly M. (2014). Measuring consciousness in severely damaged brains. Annu. Rev. Neurosci..

[5] Marino S., Bonanno L., Giorgio A. (2016). Functional connectivity in disorders of consciousness: Methodological aspects and clinical relevance. Brain Imaging Behav..

[6] Schiff N.D. (2015). Cognitive motor dissociation following severe brain injuries. JAMA Neurol..

[7] Mura E., Pistoia F., Sarà M., Sacco S., Carolei A., Govoni S. (2014). Pharmacological modulation of the state of awareness in patients with disorders of consciousness: An overview. Curr. Pharm. Des..

[8] Dehaene S., Changeux J.-P. (2011). Experimental and theoretical approaches to conscious processing. Neuron.

[9] Dehaene S., Changeux J.-P., Naccache L., Sackur J., Sergent C. (2006). Conscious, preconscious, and subliminal processing: A testable taxonomy. Trends Cogn. Sci..

[10] Tononi G. (2004). An information integration theory of consciousness. BMC Neurosci..

[11] Tononi G. (2012). The integrated information theory of consciousness: An updated account. Arch. Ital. Biol..

[12] Yeo B.T.T., Tandi J., Chee M.W.L. (2015). Functional connectivity during rested wakefulness predicts vulnerability to sleep deprivation. Neuroimage.

[13] Shao Y., Wang L., Ye E., Jin X., Ni W., Yang Y., Wen B., Hu D., Yang Z. (2013). Decreased thalamocortical functional connectivity after 36 hours of total sleep deprivation: Evidence from resting state FMRI. PLoS ONE.

[14] Miraglia F., Tomino C., Vecchio F., Gorgoni M., De Gennaro L., Rossini P.M. (2021). The brain network organization during sleep onset after deprivation. Clin. Neurophysiol..

[15] Pavlova M.K., Latreille V. (2019). Sleep disorders. Am. J. Med..

[16] Krause A.J., Simon E.B., Mander B.A., Greer S.M., Saletin J.M., Goldstein-Piekarski A.N., Walker M.P. (2017). The sleep-deprived human brain. Nat. Rev. Neurosci..

[17] Bellesi M., Haswell J.D., De Vivo L., Marshall W., Roseboom P.H., Tononi G., Cirelli C. (2018). Myelin modifications after chronic sleep loss in adolescent mice. Sleep.

[18] De Vivo L., Bellesi M., Marshall W., Bushong E.A., Ellisman M.H., Tononi G., Cirelli C. (2017). Ultrastructural evidence for synaptic scaling across the wake/sleep cycle. Science.

[19] Ulrich D. (2016). Sleep spindles as facilitators of memory formation and learning. Neural Plast..

[20] Hauglund N.L., Pavan C., Nedergaard M. (2020). Cleaning the sleeping brain–the potential restorative function of the glymphatic system. Curr. Opin. Physiol..

[21] Zada D., Bronshtein I., Lerer-Goldshtein T., Garini Y., Appelbaum L. (2019). Sleep increases chromosome dynamics to enable reduction of accumulating DNA damage in single neurons. Nat. Commun..

[22] Kusztor A., Raud L., Juel B.E., Nilsen A.S., Storm J.F., Huster R.J. (2019). Sleep deprivation differentially affects subcomponents of cognitive control. Sleep.

[23] Drummond S.P.A., Walker M., Almklov E., Campos M., Anderson D.E., Straus L.D. (2013). Neural correlates of working memory performance in primary insomnia. Sleep.

[24] Chee M.W.L., Choo W.C. (2004). Functional imaging of working memory after 24 hr of total sleep deprivation. J. Neurosci..

[25] Verweij I.M., Romeijn N., Smit D.J.A., Piantoni G., Van Someren E.J.W., van der Werf Y.D. (2014). Sleep deprivation leads to a loss of functional connectivity in frontal brain regions. BMC Neurosci..

[26] Dai X.-J., Liu C.-L., Zhou R.-L., Gong H.-H., Wu B., Gao L., Wang Y.-X.J. (2015). Long-term total sleep deprivation decreases the default spontaneous activity and connectivity pattern in healthy male subjects: A resting-state fMRI study. Neuropsychiatr. Dis. Treat..

[27] Wislowska M., Del Giudice R., Lechinger J., Wielek T., Heib D.P.J., Pitiot A., Pichler G., Michitsch G., Donis J., Schabus M. (2017). Night and day variations of sleep in patients with disorders of consciousness. Sci. Rep..

[28] Mertel I., Pavlov Y.G., Barner C., Müller F., Diekelmann S., Kotchoubey B. (2020). Sleep in disorders of consciousness: Behavioral and polysomnographic recording. BMC Med..

[29] Sebastiano D.R., Visani E., Panzica F., Sattin D., Bersano A., Nigri A., Ferraro S., Parati E., Leonardi M., Franceschetti S. (2018). Sleep patterns associated with the severity of impairment in a large cohort of patients with chronic disorders of consciousness. Clin. Neurophysiol..

[30] Blume C., Del Giudice R., Wislowska M., Lechinger J., Schabus M. (2015). Across the consciousness continuum—from unresponsive wakefulness to sleep. Front. Hum. Neurosci..

[31] Yang X., Song C., Yuan F., Zhao J., Jiang Y., Yang F., Kang X., Jiang W. (2020). Prognostic roles of sleep electroencephalography pattern and circadian rhythm biomarkers in the recovery of consciousness in patients with coma: A prospective cohort study. Sleep Med..

[32] Duclos C., Dumont M., Blais H., Paquet J., Potvin M.-J., Menon D.K., Bernard F., Gosselin N. (2014). Severe sleep-wake disturbances in acute and post-acute traumatic brain injury: A case report. Brain Inj..

[33] Arnaldi D., Terzaghi M., Cremascoli R., De Carli F., Maggioni G., Pistarini C., Nobili F., Moglia A., Manni R. (2016). The prognostic value of sleep patterns in disorders of consciousness in the sub-acute phase. Clin. Neurophysiol..

[34] Valente M., Placidi F., Oliveira A.J., Bigagli A., Morghen I., Proietti R., Gigli G.L. (2002). Sleep organization pattern as a prognostic marker at the subacute stage of post-traumatic coma. Clin. Neurophysiol..

[35] Tononi G., Cirelli C. (2014). Sleep and the price of plasticity: From synaptic and cellular homeostasis to memory consolidation and integration. Neuron.

[36] Gottshall J.L., Rossi Sebastiano D. (2020). Sleep in disorders of consciousness: Diagnostic, prognostic, and therapeutic considerations. Curr. Opin. Neurol..

[37] Sateia M.J. (2014). International classification of sleep disorders-third edition highlights and modifications. Chest.

[38] Gardani M., Morfiri E., Thomson A., O’Neill B., McMillan T.M. (2015). Evaluation of Sleep Disorders in Patients with Severe Traumatic Brain Injury During Rehabilitation. Arch. Phys. Med. Rehabil..

[39] Mathias J.L., Alvaro P.K. (2012). Prevalence of sleep disturbances, disorders, and problems following traumatic brain injury: A meta-analysis. Sleep Med..

[40] Bukhari M.A.A., Alghtani M.A.M., Sultan Z., Aljohani A.A.A., Alhazmi I.H.M. (2021). Diagnosis and treatment of sleep disorders: A brief review. Int. J. Med. Dev. Ctries..

[41] Kondziella D., Bender A., Diserens K., van Erp W., Estraneo A., Formisano R., Laureys S., Naccache L., Ozturk S., Rohaut B. (2020). European Academy of Neurology guideline on the diagnosis of coma and other disorders of consciousness. Eur. J. Neurol..

[42] Ford M.E., Groet E., Daams J.G., Geurtsen G.J., Van Bennekom C.A.M., Van Someren E.J.W. (2020). Non-pharmacological treatment for insomnia following acquired brain injury: A systematic review. Sleep Med. Rev..

[43] Pilon L., Frankenmolen N., Bertens D. (2021). Treatments for sleep disturbances in individuals with acquired brain injury: A systematic review. Clin. Rehabil..

[44] Moher D., Liberati A., Tetzlaff J., Altman D.G., Group P. (2009). Preferred reporting items for systematic reviews and meta-analyses: The PRISMA statement. PLoS Med..

[45] Landis J.R., Koch G.G. (1977). The measurement of observer agreement for categorical data. Biometrics.

[46] Adams Z.M., Forgacs P.B., Conte M.M., Nauvel T.J., Drover J.D., Schiff N.D. (2016). Late and progressive alterations of sleep dynamics following central thalamic deep brain stimulation (CT-DBS) in chronic minimally conscious state. Clin. Neurophysiol..

[47] Formica F., Pozzi M., Avantaggiato P., Molteni E., Arrigoni F., Giordano F., Clementi E., Strazzer S. (2017). Disordered consciousness or disordered wakefulness? The importance of prolonged polysomnography for the diagnosis, drug therapy, and rehabilitation of an unresponsive patient with brain injury. J. Clin. Sleep Med..

[48] Gottshall J.L., Adams Z.M., Forgacs P.B., Schiff N.D. (2019). Daytime central thalamic deep brain stimulation modulates sleep dynamics in the severely injured brain: Mechanistic insights and a novel framework for alpha-delta sleep generation. Front. Neurol..

[49] Locatelli F., Formica F., Galbiati S., Avantaggiato P., Beretta E., Carnovale C., Pozzi M., Clementi E., Strazzer S. (2019). Polysomnographic analysis of a pediatric case of baclofen-induced central sleep apnea. J. Clin. Sleep Med..

[50] Silva M.A., Schwartz D.J., Nakase-Richardson R. (2019). Functional improvement after severe brain injury with disorder of consciousness paralleling treatment for comorbid obstructive sleep apnoea: A case report. Int. J. Rehabil. Res..

[51] Blume C., Lechinger J., Santhi N., Del Giudice R., Gnjezda M.-T., Pichler G., Scarpatetti M., Donis J., Michitsch G., Schabus M. (2017). Significance of circadian rhythms in severely brain-injured patients: A clue to consciousness?. Neurology.

[52] Dhamapurkar S.K., Wilson B.A., Rose A., Watson P., Shiel A. (2017). Does Modafinil improve the level of consciousness for people with a prolonged disorder of consciousness? A retrospective pilot study. Disabil. Rehabil..

[53] Cologan V., Drouot X., Parapatics S., Delorme A., Gruber G., Moonen G., Laureys S. (2013). Sleep in the unresponsive wakefulness syndrome and minimally conscious state. J. Neurotrauma.

[54] Yaron I., Melloni L., Pitts M., Mudrik L. (2021). How are theories of consciousness empirically tested? The Consciousness Theories Studies (ConTraSt) database. J. Vis..

[55] Chemelli R.M., Willie J.T., Sinton C.M., Elmquist J.K., Scammell T., Lee C., Richardson J.A., Williams S.C., Xiong Y., Kisanuki Y. (1999). Narcolepsy in orexin knockout mice: Molecular genetics of sleep regulation. Cell.

[56] Pack A.I., Black J.E., Schwartz J.R.L., Matheson J.K. (2001). Modafinil as adjunct therapy for daytime sleepiness in obstructive sleep apnea. Am. J. Respir. Crit. Care Med..

[57] Moldofsky H., Broughton R.J., Hill J.D. (2000). A randomized trial of the long-term, continued efficacy and safety of modafinil in narcolepsy. Sleep Med..

[58] Czeisler C.A., Walsh J.K., Roth T., Hughes R.J., Wright K.P., Kingsbury L., Arora S., Schwartz J.R.L., Niebler G.E., Dinges D.F. (2005). Modafinil for excessive sleepiness associated with shift-work sleep disorder. N. Engl. J. Med..

[59] Roth T., Schwartz J.R.L., Hirshkowitz M., Erman M.K., Dayno J.M., Arora S. (2007). Evaluation of the safety of modafinil for treatment of excessive sleepiness. J. Clin. Sleep Med..

[60] Jasinski D.R., Kovacevic-Ristanovic R. (2000). Evaluation of the abuse liability of modafinil and other drugs for excessive daytime sleepiness associated with narcolepsy. Clin. Neuropharmacol..

[61] Lees J., Michalopoulou P.G., Lewis S.W., Preston S., Bamford C., Collier T., Kalpakidou A., Wykes T., Emsley R., Pandina G. (2017). Modafinil and cognitive enhancement in schizophrenia and healthy volunteers: The effects of test battery in a randomised controlled trial. Psychol. Med..

[62] Rasmussen N.-A., Schrøder P., Olsen L.R., Brødsgaard M., Undén M., Bech P. (2005). Modafinil augmentation in depressed patients with partial response to antidepressants: A pilot study on self-reported symptoms covered by the Major Depression Inventory (MDI) and the Symptom Checklist (SCL-92). Nord. J. Psychiatry.

[63] Turner D.C., Robbins T.W., Clark L., Aron A.R., Dowson J., Sahakian B.J. (2003). Cognitive enhancing effects of modafinil in healthy volunteers. Psychopharmacology.

[64] Mahoney J.J. (2019). Cognitive dysfunction in individuals with cocaine use disorder: Potential moderating factors and pharmacological treatments. Exp. Clin. Psychopharmacol..

[65] Kalechstein A.D., Mahoney III J.J., Yoon J.H., Bennett R., De La Garza II R. (2013). Modafinil, but not escitalopram, improves working memory and sustained attention in long-term, high-dose cocaine users. Neuropharmacology.

[66] Esposito R., Cilli F., Pieramico V., Ferretti A., Macchia A., Tommasi M., Saggino A., Ciavardelli D., Manna A., Navarra R. (2013). Acute effects of modafinil on brain resting state networks in young healthy subjects. PLoS ONE.

[67] Ciurleo R., Bramanti P., Calabrò R.S. (2013). Pharmacotherapy for disorders of consciousness: Are ‘awakening’drugs really a possibility?. Drugs.

[68] Georgiopoulos M., Katsakiori P., Kefalopoulou Z., Ellul J., Chroni E., Constantoyannis C. (2010). Vegetative state and minimally conscious state: A review of the therapeutic interventions. Stereotact. Funct. Neurosurg..

[69] Giacino J.T., Whyte J., Bagiella E., Kalmar K., Childs N., Khademi A., Eifert B., Long D., Katz D.I., Cho S. (2012). Placebo-controlled trial of amantadine for severe traumatic brain injury. N. Engl. J. Med..

[70] Noormandi A., Shahrokhi M., Khalili H. (2017). Potential benefits of zolpidem in disorders of consciousness. Expert Rev. Clin. Pharmacol..

[71] Thibaut A., Schiff N., Giacino J., Laureys S., Gosseries O. (2019). Therapeutic interventions in patients with prolonged disorders of consciousness. Lancet Neurol..

[72] Margetis K., Korfias S.I., Gatzonis S., Boutos N., Stranjalis G., Boviatsis E., Sakas D.E. (2014). Intrathecal baclofen associated with improvement of consciousness disorders in spasticity patients. Neuromodulation Technol. Neural Interface.

[73] Pistoia F., Sacco S., Sara M., Franceschini M., Carolei A. (2015). Intrathecal Baclofen: Effects on Spasticity, Pain, and Consciousness in Disorders of Consciousness and Locked-in Syndrome. Curr. Pain Headache Rep..

[74] Castronovo V., Scifo P., Castellano A., Aloia M.S., Iadanza A., Marelli S., Cappa S.F., Strambi L.F., Falini A. (2014). White matter integrity in obstructive sleep apnea before and after treatment. Sleep.

[75] Arendt J., Broadway J. (1987). Light and melatonin as zeitgebers in man. Chronobiol. Int..

[76] Ancoli-Israel S., Gehrman P., Martin J.L., Shochat T., Marler M., Corey-Bloom J., Levi L. (2003). Increased light exposure consolidates sleep and strengthens circadian rhythms in severe Alzheimer’s disease patients. Behav. Sleep Med..

[77] Beaven C.M., Ekström J. (2013). A comparison of blue light and caffeine effects on cognitive function and alertness in humans. PLoS ONE.

[78] Videnovic A., Klerman E.B., Wang W., Marconi A., Kuhta T., Zee P.C. (2017). Timed light therapy for sleep and daytime sleepiness associated with Parkinson disease: A randomized clinical trial. JAMA Neurol..

[79] Cajochen C. (2007). Alerting effects of light. Sleep Med. Rev..

[80] Kundu B., Brock A.A., Englot D.J., Butson C.R., Rolston J.D. (2018). Deep brain stimulation for the treatment of disorders of consciousness and cognition in traumatic brain injury patients: A review. Neurosurg. Focus.

[81] Royal College of Physicians (2020). Prolonged Disorders of Consciousness Following Sudden Onset Brain Injury: National Clinical Guidelines.

[82] Giacino J.T., Katz D.I., Schiff N.D., Whyte J., Ashman E.J., Ashwal S., Barbano R., Hammond F.M., Laureys S., Ling G.S.F. (2018). Practice Guideline Update Recommendations Summary: Disorders of Consciousness: Report of the Guideline Development, Dissemination, and Implementation Subcommittee of the American Academy of Neurology; the American Congress of Rehabilitation Medicine; and the National Institute on Disability, Independent Living, and Rehabilitation Research. Arch. Phys. Med. Rehabil..

[83] Pratap-Chand R., Gourie-Devi M. (1985). Bruxism: Its significance in coma. Clin. Neurol. Neurosurg..

[84] Pennestri M.H., Montplaisir J., Colombo R., Lavigne G., Lanfranchi P.A. (2007). Nocturnal blood pressure changes in patients with restless legs syndrome. Neurology.

[85] Galbiati A., Marelli S., Giora E., Zucconi M., Oldani A., Ferini-Strambi L. (2015). Neurocognitive function in patients with idiopathic Restless Legs Syndrome before and after treatment with dopamine-agonist. Int. J. Psychophysiol..

[86] de Alencar N.A., Leão C.S., Leão A.T.T., Luiz R.R., Fonseca-Gonçalves A., Maia L.C. (2017). Sleep bruxism and anxiety impacts in quality of life related to oral health of Brazilian children and their families. J. Clin. Pediatr. Dent..

[87] Silvestri R., Gagliano A., Aricò I., Calarese T., Cedro C., Bruni O., Condurso R., Germanò E., Gervasi G., Siracusano R. (2009). Sleep disorders in children with Attention-Deficit/Hyperactivity Disorder (ADHD) recorded overnight by video-polysomnography. Sleep Med..

[88] Frohlich J., Toker D., Monti M.M. (2021). Consciousness among delta waves: A paradox. Brain.

[89] Scheinin A., Kantonen O., Alkire M., Långsjö J., Kallionpää R.E., Kaisti K., Radek L., Johansson J., Sandman N., Nyman M. (2021). Foundations of human consciousness: Imaging the twilight zone. J. Neurosci..

