# Supplementary material for: Sleep Treatments in Disorders of Consciousness: A Systematic Review
Source: Diagnostics (Basel). 2021 Dec 31;12(1):88. doi: 10.3390/diagnostics12010088 (PMC8775271; doi:10.3390/diagnostics12010088)
Supplement: Supplementary file 1 [file diagnostics-12-00088-s001.zip › diagnostics-1510293-supplementary.pdf]

Supplemental materials

Sleep treatments in Disorders of Consciousness: A systematic review

Cacciatore M.[1], Magnani F.G.[1]*, Leonardi M.[1], Rossi Sebastiano D.[2], Sattin D.[3]

[1] UOC Neurologia, Salute Pubblica, Disabilità; Fondazione IRCCS Istituto Neurologico Carlo Besta, Milan, Italy

[2] Unita` di Neurofisiopatologia, Fondazione-IRCCS-Istituto Neurologico Carlo Besta, Milan, Italy

[3] IRCCS Istituti Clinici Scientifici Maugeri di Milano, 20138 Milan, Italy

* Correspondence: Dr. Magnani Francesca Giulia, PhD; UOC Neurologia, Salute Pubblica, Disabilità; Fondazione IRCCS Istituto Neurologico Carlo Besta, Milan, Italy; e-mail: francesca.magnani@istituto-besta.it; phone: +39 02 23942188

Search strategy

MEDLINE (advanced search)

#1 (search string related to the domain of disorders of consciousness)

((((((((((((((((((((((((Persistent Vegetative State[MeSH Terms]) OR (Consciousness Disorders[MeSH Terms]))OR (Persistent Vegetative State[Title/Abstract])))) OR (Persistent Vegetative States[Title/Abstract]) OR (Consciousness Disorders[Title/Abstract])) OR (Consciousness Disorder[Title/Abstract])) OR (Vegetative State[Title/Abstract])) OR (Vegetative States[Title/Abstract])) OR (Unawareness State[Title/Abstract])) OR (Unawareness States[Title/Abstract])) OR (Minimally Conscious State[Title/Abstract])) OR (Minimally Conscious States[Title/Abstract])) OR (Unresponsive wakefulness syndrome[Title/Abstract])) OR (Disorders of Consciousness[Title/Abstract])))) OR (Disorder of Consciousness[Title/Abstract])) OR ('severe traumatic brain injury'[Title/Abstract]))) OR ("brain injury"[Title/Abstract])) OR ("hemorrhagic brain injury")) OR (ischemic brain injury)) OR ("post anoxic brain injury")) OR ("traumatic brain injury")))

#2 (search string related to the domain of sleep disorders)

(((((((((((((((((((((((((((((((Sleep Disorders, Intrinsic[MeSH Terms]) OR (Sleep Bruxism[MeSH Terms])) OR (Sleep Apnea, Central[MeSH Terms])) OR (Sleep Apnea, Obstructive[MeSH Terms])) OR (Sleep Wake Disorders[MeSH Terms])) OR (Sleep Apnea Syndromes[MeSH Terms])) OR (Restless Legs Syndrome[MeSH Terms])) OR (Sleep Disorder[Title/Abstract])) OR (Sleep Disorders[Title/Abstract])) OR (Bruxism[Title/Abstract])) OR (Bruxisms[Title/Abstract])) OR (Teeth Grinding Disorder[Title/Abstract])) OR (Central Sleep Apneas[Title/Abstract])) OR (Sleep

Apnea[Title/Abstract])) OR (Obstructive Sleep Apneas[Title/Abstract])) OR (Obstructive Sleep Apnea[Title/Abstract]))) OR (Sleep-Disordered Breathing[Title/Abstract])) OR (Apnea Syndrome[Title/Abstract])) OR (Apnea Syndromes[Title/Abstract])) OR (Hypopnea, Sleep[Title/Abstract]) OR (Hypopneas, Sleep[Title/Abstract]) OR (Restless Legs[Title/Abstract])) OR (Restless Leg Syndrome[Title/Abstract])) OR (Willis Ekbom Disease[Title/Abstract])) OR (Wittmaack-Ekbom Syndrome[Title/Abstract]))) OR (periodic limb movement disorder[Title/Abstract])) OR (periodic limb movement disorders[Title/Abstract])) OR (nocturnal myoclonus syndrome[Title/Abstract])))))

#3 (search string related to the domain of treatment)

'treatment outcome'/exp/mj OR 'treatment outcome'/exp OR 'treatment outcome' OR 'therapy'/exp/mj OR 'therapy'/exp OR 'therapy' OR 'therapeutic':ti,ab,kw OR 'outcome treatment':ti,ab,kw OR 'clinical effectiveness':ti,ab,kw OR 'treatment effectiveness':ti,ab,kw OR 'disease management':ti,ab,kw OR ('treatment' NEAR/4 ('sleep' OR 'apnea*' OR 'bruxism'))

#1 AND #2 AND #3

SCOPUS (advanced search)

#1

(TITLE-ABS-KEY ("Consciousness Disorders*") OR TITLE-ABS-KEY ("Persistent Vegetative State*") OR TITLE-ABS-KEY ("Vegetative State*") OR TITLE-ABS-KEY ("Unresponsive wakefulness syndrome") OR TITLE-ABS-KEY ("Minimally Conscious State*") OR TITLE-ABS-KEY ("Disorders of Consciousness*") OR TITLE-ABS-KEY ("severe traumatic brain injury") OR TITLE-ABS-KEY ("brain injury") OR ALL ("hemorrhagic brain injury") OR ALL ("ischemic brain injury") OR ALL ("post anoxic brain injury") OR ALL ("traumatic brain injury"))

#2

(TITLE-ABS-KEY ("Sleep Disorder*") OR TITLE-ABS-KEY ("Sleep Bruxism") OR TITLE-ABS-KEY (" Central sleep apnea*") OR TITLE-ABS-KEY ("obstructive sleep apnea*") OR TITLE-ABS-KEY ("Sleep Apnea Syndrome*") OR TITLE-ABS-KEY (bruxism*) OR TITLE-ABS-KEY ("Teeth Grinding Disorder") OR TITLE-ABS-KEY ("Sleep-Disordered Breathing") OR TITLE-ABS-KEY ("Hypopnea*") OR TITLE-ABS-KEY ("Restless Leg Syndrome") OR TITLE-ABS-KEY ("Ekbom Disease") OR TITLE-ABS-KEY ("periodic limb movement disorder*") OR TITLE-ABS-KEY ("nocturnal myoclonus syndrome") OR TITLE-ABS-KEY ("Restless Leg*") OR TITLE-ABS-KEY ("Sleep Wake Disorder*"))

#3

(TITLE-ABS-KEY ("treatment outcome") OR TITLE-ABS-KEY ("therapy") OR TITLE-ABS-KEY ("therapeutic") OR TITLE-ABS-KEY ("outcome treatment") OR TITLE-ABS-KEY ("disease management"))

#1 AND #2 AND #3

EMBASE (advanced search)

#1

'consciousness disorder'/exp/mj OR 'consciousness disorder' OR 'persistent vegetative state'/exp/mj OR 'persistent vegetative state' OR 'vegetative stage':ti,ab,kw OR 'vegetative stages':ti,ab,kw OR 'minimally conscious state':ti,ab,kw OR 'minimally conscious states':ti,ab,kw OR 'disorder of consciousness':ti,ab,kw OR 'disorders of consciousness':ti,ab,kw OR 'unresponsive wakefulness syndrome':ti,ab,kw OR 'severe traumatic brain injury':ti,ab,kw OR 'brain injury':ti,ab,kw OR 'hemorrhagic brain injury' OR 'ischemic brain injury' OR 'post anoxic brain injury' OR 'traumatic brain injury'

#2

'sleep disorder'/exp/mj OR 'sleep disorder' OR 'central sleep apnea syndrome'/exp/mj OR 'central sleep apnea syndrome' OR 'sleep bruxism'/exp/mj OR 'sleep bruxism' OR 'sleep disordered breathing'/exp/mj OR 'sleep disordered breathing' OR 'sleep apnea'/exp/mj OR 'sleep apnea' OR 'restless legs syndrome'/exp/mj OR 'restless legs syndrome' OR 'obstructive sleep apnea'/exp/mj OR 'obstructive sleep apnea' OR 'breathing disorder*':ti,ab,kw OR 'sleep hypopnoea*':ti,ab,kw OR 'hypopnoea*':ti,ab,kw OR bruxism*:ti,ab,kw OR 'restless leg':ti,ab,kw OR 'ekbom syndrome':ti,ab,kw OR 'periodic limb movement disorder*':ti,ab,kw OR 'nocturnal myoclonus syndrome':ti,ab,kw OR 'sleep wake disorder*':ti,ab,kw

#3

'treatment outcome'/exp/mj OR 'treatment outcome'/exp OR 'treatment outcome' OR 'therapy'/exp/mj OR 'therapy'/exp OR 'therapy' OR 'therapeutic':ti,ab,kw OR 'outcome treatment':ti,ab,kw OR 'clinical effectiveness':ti,ab,kw OR 'treatment effectiveness':ti,ab,kw OR 'disease management':ti,ab,kw OR ('treatment' NEAR/4 ('sleep' OR 'apnea*' OR 'bruxism'))

#1 AND #2 AND #3

Web of Science (advanced search)

#1

TS=('consciousness disorder*') OR TS=('persistent vegetative state*') OR TS=('vegetative state*') OR TS=('minimally conscious state*') OR TS=('Unresponsive wakefulness syndrome') OR TS=("disorder of consciusness*") OR TS=('severe traumatic brain injury') OR ALL=('brain injury') OR ALL=('hemorrhagic brain injury') OR ALL=("ischemic brain injury") OR ALL=("post anoxic brain injury") OR ALL=("traumatic brain injury")

#2

TS=('sleep disorder*') OR TS=('Sleep Bruxism') OR TS=('Central sleep apnea*') OR TS=('obstructive sleep apnea*') OR TS=('Sleep Apnea Syndrome*') OR TS=('bruxism*') OR TS=('Teeth Grinding Disorder') OR TS=('Sleep-Disordered Breathing') OR TS=('Hypopnea*') OR TS=('Restless Leg Syndrome') OR TS=('Ekbom Disease') OR TS=('periodic limb movement disorder*') OR TS=('Sleep Wake Disorder*')

#3

TS=('treatment outcome') OR TS=('therapy') OR TS=('therapeutic') OR TS=('disease management') OR TS=('treatment effectiveness')

#1 AND #2 AND #3

CINAHL COMPLETE (advanced search)

#1

"consciousness disorder*" OR "persistent vegetative state*" OR "vegetative state*" OR "minimally conscious state*" OR "Unresponsive wakefulness syndrome" OR "disorder of consciusness*" OR "severe traumatic brain injury" OR "hemorrhagic brain injury" OR "ischemic brain injury" OR "post anoxic brain injury" OR "traumatic brain injury"

#2

"sleep disorder*" OR "Sleep Bruxism" OR "Central sleep apnea*" OR "obstructive sleep apnea*" OR "Sleep Apnea Syndrome*" OR bruxism* OR "Teeth Grinding Disorder" OR "Sleep-Disordered Breathing" OR "Hypopnea*" OR "periodic limb movement disorder*" OR "Restless Leg Syndrome" OR "Sleep Wake Disorder*"

#3

"treatment outcome" OR therapy* OR therapeutic OR treatment* OR "treatment effectiveness" OR "disease management"

#1 AND #2 AND #3
